# Supplementary material for: Identification of photoperiod-induced specific miRNAs in the adrenal glands of Sunite sheep (Ovis aries)
Source: Front Vet Sci. 2022 Jul 22;9:888207. doi: 10.3389/fvets.2022.888207 (PMC9354845; doi:10.3389/fvets.2022.888207)
Supplement: Supplementary file 1 [file Data_Sheet_1.ZIP › Supplementary file/miRDeep2 file.zip!/miRDeep2 file/Novel_Structure/LP42A2/Novel_660.pdf]

Provisional ID	:	NC_019481.2_35030
Score total	:	1
Score for star read(s)	:	-1.3
Score for read counts	:	0
Score for mfe	:	0.7
Score for randfold	:	1.6
Score for cons. seed	:	
Total read count	:	38
Mature read count	:	38
Loop read count	:	0
Star read count	:	0

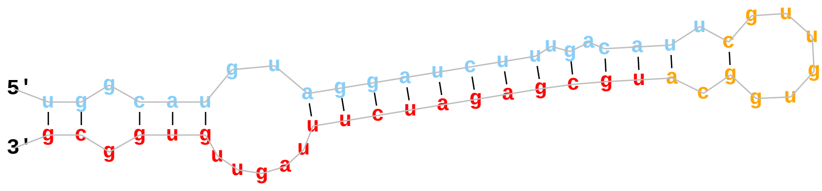

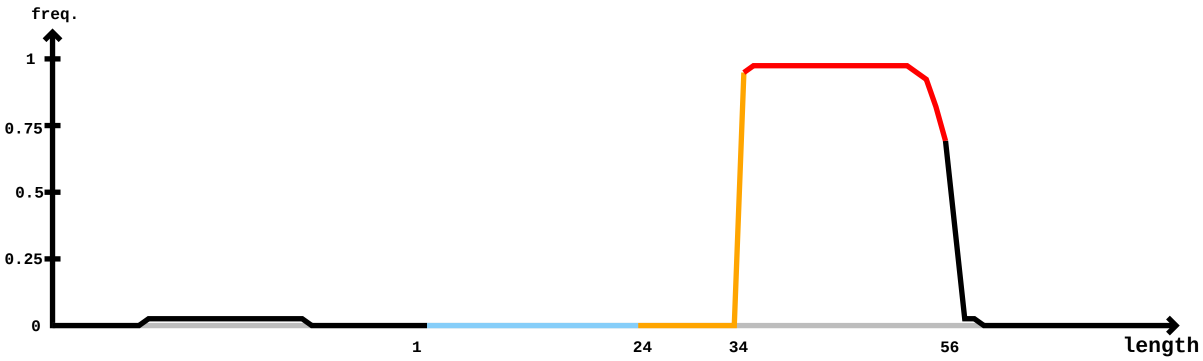

Star Mature

```
5'- gaguuuuuauuuauuuggcuguaccaggucuuaguuguggcauguaggaucuuugacauucguuguggcaugcgagaucuuuaguuguggcguaugaauucuugguugcagcaca   -3'    exp
    .................(((((((((((..(((..(((.(((...((((((((.(.(((.(......).))))))))))))....))).)))..)))...))))).))))))...     reads      mm          sample
    ........auuuUuuuggcuguacc..........................................................................................     1          1           seq
    ......................................................................ugcgagaucuuuaguugC...........................     1          1           seq
    ......................................................................ugcgagaucuuuaguugug..........................     1          0           seq
    ......................................................................ugcAagaucuuuaguugugg.........................     1          1           seq
    ......................................................................ugcgagaucuuuaguugugg.........................     3          0           seq
    ......................................................................ugcAagaucuuuaguuguggc........................     2          1           seq
    ......................................................................ugcgagaucuuuaguuguggc........................     3          0           seq
    ......................................................................ugcCagaucuuuaguuguggcg.......................     1          1           seq
    ......................................................................ugcgagaucuuuaguuguggcg.......................     14         0           seq
    ......................................................................ugcgagaucuuuaguuguggcA.......................     6          1           seq
    ......................................................................ugcgagaucuuuaguuguggcgu......................     4          0           seq
    ......................................................................ugcgagaucuuuaguuguggcguau....................     1          0           seq
    .......................................................................gcgagaucuuuaguuguggcgu......................     1          0           seq
```
